# Extracts from a Paper upon the Pathological Relations of the Gastric and Intestinal Tubes*Read before the New York County Medical Society, February 13, 1871.

**Published:** 1871-03

**Authors:** Austin Flint

**Affiliations:** Professor of the Principles and Practice of Medicine, of Clinical Medicine, in the Bellevue Hospital Medical College


### Miscellaneous.

### Extracts from a paper upon the Pathological Relations of the Gastric and Intestinal Tubes.*

By AUSTIN FLINT, M. D., Professor of the Principles and Practice of Medicine, of Clinical Medicine, in the Bellevue Hospital Medical College.

The paper which I am about to read will not contain any original observations relating to the morbid anatomical changes taking place in the gastric and intestinal tubules. Experience, together with proficiency in practical microscopy, is requisite for the study of these changes, and I have not the qualifications nor the leisure to engage in it. As regards the histological aspect of my subject, the facts to which I refer have been contributed exclusively by three English observers, namely, Handfield Jones, Wilson Fox, and Samuel Fenwick. Aside from these facts, the considerations which I shall present are those arising from a clinical and physiological point of view. I have for many years had a strong conviction that the secretory glands of the alimentary canal constitute a territory in pathology, until lately almost a *terra incognita*, whence are to be derived very important additions to our knowledge of disease. It

* Read before the New York County Medical Society, February 13, 1871.

has seemed to me a rational supposition that destructive lesions of these glands exist in a class of cases characterized by progressive and fatal inanition taking place without the evidence of disease existing elsewhere sufficiently to explain the symptoms and the termination. I have long been accustomed in lectures and consultations, as also in published writings, to predict that, in the cases now referred to, the tubules of the alimentary canal will be found to be the seat of degenerative changes which, in view of the important functions of these glands, will account satisfactorily for the symptomatic phenomena and death. I propose, in the first place, togive a brief account of some of the cases of this class which have fallen under my observation. After having done this, I will refer to the histological facts contributed by the three English observers whomI have named; and I will conclude by offering some considerations relating to the importance of the study of gastric and intestinal tubules, not only in the cases in which, as may be conjectured, the primary and essential disease is here seated, but with reference to the probable occurrence in this situation of morbid changes which enter more or less largely into the pathology of a diversity of diseases:

Case. I.—Fourteen years ago I had the care of a patient, about sixty years of age, whom I had known intimately for twenty years. During all this period and previously his health had been excellent, with the exception of occasional attacks of gout. He was a man of large frame, and his muscular system was largely developed; he had superior mental endowments, and his habits of life were unexceptionable. The only apparent cause of disease was a certain amount of mental annoyance incident to some domestic troubles. This patient, who had always been a good feeder, found his desire for food diminishing, and, for several months, he gradually reduced his diet in accordance with a progressive diminution of appetite, not suspecting that he had any disease, until his food consisted chiefly of liquids. His weight diminished, without notable emaciation; his strength failed, and at length he was obliged to keep the bed. At this juncture he came under my care. He had now complete anorexia, and a repugnance to food to such an extent that he could scarcely be induced to take it in any form. He was unable to get up without assistance, there being no paralysis, but only great muscular weakness. An examination of all the organs failed to show any evidence of disease anywhere. The heart and lungs were sound. The urine was free from changes denoting disease of the kidneys. Nothing was discovered on exploration of the abdomen. The intellect remained intact, except that, for a day or two before death, there was, at times, some mental aberration. The pulse and the skin did not denote fever. Death took place evidently from inanition, the mode of dying being purely by asthenia. Various tonic remedies and stimulants were given, with no benefit. There was no autopsy. The case was one in which I felt an unusual interest and responsibility. I studied it carefully, and I was wholly unable to form any definite idea of the nature and seat of the disease.

Case II.—In March, 1866, I visited at New Bedford, Mass., in consultation with Dr. Wm. A. Gordon, a gentleman, aged sixty. He had always been a healthy man up to some months before I saw him. Without any appreciable cause, he had gradually lost desire for food, and at length he had a repugnance to every form of aliment. *Pari passu* with the increase of anorexia, his muscular strength diminished, and at the time of my visit he was confined to the bed. It was impossible, in this case, to discover any head or general disease. There were not present symptoms, other than the inability to take food. going to show disease of the stomach or any of the digestive organs. The condition of the organs within the chest was normal. The urine, which was repeatedly examined, gave no evidence of renal disease. The intellectual faculties were unimpaired. There was no febrile movement. He had been seen by Dr. Bowditch, of Boston. Death took place not long after my visit, the immediate cause being evidently inanition. Owing to the objection of friends, there was no *post-mortem* examination. The patient in this case was a man in easy circumstances; his habits of life were, in all respects, apparently those conducive to health and long life. His mind was occupied with business without undue strain or excitement; he lived well, but was perfectly temperate both in eating and drinking, and his temper was remarkably equable. There was no apparent causation of the unknown fatal disease.

CASE III.—In the summer of 1868 I saw repeatedly with my late lamented colleague, Dr. George T. Elliot, a gentleman of this city, aged about fifty-five years. He was a wealthy, retired merchant, greatly esteemed for his ability and probity. His habits were in all respects temperate and regular. He had a placid and benevolent disposition. To all appearance, his life might be cited as a model of practical hygene. He had aways had good heath until some months before I saw him, when his appetite had begun to decline, and he frequently vomited after eating. In this case the heart, lungs, and liver, were carefully interrogated, and no evidence of disease of these organs was discovered. Nothing was found on careful exploration of the abdomen. Attention was directed particularly to the stomach on account of the occasional vomiting, but there was no symptoms denoting either cancer, ulcer or gastritis. It seemed to us that the inability to take, retain, and digest food, alone stood in the way of the recovery of health. Various tonics were tried, and change of air, with apparently some temporary benefit. I saw him again in 1869. A repetition of the examination with reference to the existence of some definite disease was negative, as before. He was now quite weak, keeping the bed most of the time. During the last few weeks of his life I did not see him. He was under the care of a homoeopathic practitioner; but I have ascertained that there was no essential change in the symptoms, and that the mode of dying was by exhaustion.

CASE IV.—In may, 1869, an analogous case came under my observation. The patient was under the care of Dr. John C. Hutchinson, of Brooklyn. Dr. Willard Parker was associated in consultation. In this case the age was about sixty years. There had been, for several months, progressive diminution of appetite and strength. The patient, when I saw him, was confined to the bed, and death took place shortly afterward. Looseness of the bowels was a feature in this case, but the history and symptoms denoted intestinal indigestion, and not either intestinal ulceration or inflammation. All the vital organs, in as far as they could be interrogated, seemed to be free from disease. Emaciation was more marked in this case than in the preceding cases.

In these four cases the patients were males. I have, however, met analogous cases in females. In October, 1868, I visited, in consultation with my colleague, Dr. Fordyce Barker, a female patient, and another female patient in January, 1869; the facts in both being essentially the same as in the cases in which I have given a brief summary. The age in each of Dr. Barker's cases was in the neighborhood of sixty years.

These cases represent a class, examples of which, I am sure, all practicing physicians of much experience must have met with. The marked characteristics are gradual and at length complete loss of appetite and digestive power, with progressive debility, and death by exhaustion, adequate morbid condition seated elsewhere than in the glands of the alimentary canal being excluded by an investigation of the symptoms and signs. In the cases just cited the patients were somewhat advanced in years, that is, near the age of sixty; they were healthy prior to the illness, which ended fatally; this illness was developed imperceptibly, and advanced slowly; the persons were singularly exempt from apparent morbific influences, being temperate and their habits of life regular; they were in easy circumstances, and, in a great measure, withdrawn from active occupation; the intellectual faculties remained intact up to a few hours before death, and the mode of dying was typical of æsthenia.

The impairment of appetite was the first symptom. I have failed to note the kinds of food against which especially the appetite rebelled. In one of the cases I recollect distinctly that the patient ceased taking animal food in any form, and confined himself to gruel and milk-porridge for months before he was obliged to keep the bed. I think that, in the other cases, the antipathy was greatest toward the albuminoid articles of diet. This is a point of interest as bearing on the localization of the affection in either the gastric or the intestinal tubules separately, and on the predominance of the affection in either, if both sets of glands be affected.

My object thus far has been to show, from a clinical point of view, *first*, the existence of a well-defined class of cases characterized by anorexia, impaired digestion, progressive debility, and death from inanition; and, *second*, in view of these clinical characteristics, together with the absence of adequate lesions elsewhere, the probability that the essential disease is seated in the secretory glands of the alimentary canal. Now let me ask what is wanted in order that, in this class of cases, lesions of these glands, involving loss of their functional capacity, shall be entitled to be recognized as an established fact? The answer to this question is obvious. The existence of lesions, more or less extensive and destructive, must be demonstrated by microscopical examinations after death. The abnormal changes must be shown to be morbid, that is, not cadaveric; and their constancy in this class of cases must be established by a sufficient number of microscopical examinations. As I stated at the outset, I have no original observations to offer in relation to these points of investigation. An important contribution, however, has been made recently by Dr. Samuel Fenwick, of London. Dr. Fenwick has demonstrated extensive and destructive lesions of the gastric tubules in a well-marked case belonging in the class the clinical characteristics of which I have considered. As Dr. Fenwick's communication led to the selection of the subject of this paper, and embraces, as I suppose, the first and only case on record in which the diagnosis of disease of the gastric tubules has been made and verified autopsically, I shall quote, without any omission, all which relates to the history of the case. The communication is contained in the London *Lancet*, the number for July 16, 1870.

It is fair to estimate the probable importance of the pathological relations of the secreting glands of the alimentary mucous membrane by physiological importance. The tubules of the stomach and the intestines together form an immense glandular apparatus. The functional activity of the gastric tubules is known to be extremely great, experiments appearing to show that the gastric juice secreted during twenty-four hours amounts to the enormous quantity of fourteen pounds, being not much less than the avarage quantity of blood contained in the body. The tubules in the intestine, owing to the greater extent of the mucous surface, exceed in number vastly those of the stomach, but experiments have as yet failed to furnish any data for determining the quantity of the intestinal juice. The amount of the latter, perhaps, exceeds that of the gastric juice, in a proportion corresponding to the difference as regards the number of tubules. These two digestive liquids, together with the salivary fluids, the bile, and the pancreatic secretion, are at the portals of vegetative or organic life. They are essential as the first of the series of processes by which aliment is converted into the blood and the tissues. Upon gastric and intestinal digestion depend the consecutive functions ending in growth and repair, as also the normal condition of all the organs of the body. These physiological facts are trite enough, but they lead to a consideration which, it is evident, has not received sufficient attention, namely, the glands which secrete the essential factors in digestion cannot be extensively diseased without giving rise to impoverished blood, impaired nutrition, diminished muscular strength, weakness of the mental powers, and various abnormal conditions incident to these effects. In cases in which the glands are alone diseased, it is plain that their secretory action may be so far affected as to cause death from inanition; and, of course, the evils and the dangers arising from extensive disease of these glands are enhanced in proportion to the gravity of other associated affections.

Another consideration, pointing to the probable importance of the pathological relations of the gastric and intestinal tubules, is the difficulty, in many cases of different diseases, of explaining certain symptoms and accounting for death otherwise than by supposing these glands to be diseased. Fenwick raises this point in his memoir on morbid changes in the stomach and intestinal villi in persons dying with cancer of the breast. He says: "In many cases of cancer we can scarcely account for the death of the patient. There is no secondary formation in any important organ, and the failure in strength has been out of proportion to the amount of local mischief, so that we are forced to admit either that the blood has been inffected, or that some fatal change of a non-cancerous nature has occurred in the viscera." The statement is equally true of various other affections. Cases of phthisis furnish illustrations with which every one is familiar. How common is it to see on the one hand persons living on for months and years with enormous destruction of the pulmonary organs, and, on the other hand, persons dying with comparatively a small amount of damage! We say of the latter cases, that the vital powers soon give way, and, of the former cases, that there is a remarkable tolerance of disease. We express in this way obvious clinical facts, but we do not explain them. Clinical observation teaches that in cases of phthisis, and also in other chronic affections, the speedy giving way of the vital powers, on the one hand, or, on the other hand, the remarkable tolerance, depends, other things being equal, on the ability or otherwise to ingest, digest, and appropriate food. This clinical fact suggests the question, Why is it that, with an equal amount of disease of the lungs, or elsewhere, in some cases there is a loss of appetite, of digestion, and consequently of appropriation, leading to exhaustion and death, while, in other cases, these functions being preserved, the vital powers are maintained and life continues? Taking into view the observations of Jones, Fox, and Fenwick. which show that in phthisis and other chronic affections the glands secreting the digestive fluids are liable to become diseased, the answer to this question is that, probably, in the one class of cases these glands are but little or not at all affected, while in the other class of cases they are the seat of morbid changes.

Reasoning from a physiological stand-point, and also, to the extent of our present knowledge, from clinical and autopsical observations, we are warranted in considering a persistent loss or impairment of digestive power, together with anorexia, as the symptomatic evidence of morbid changes in the gastric and intestinal tubules. Extensive destruction of these glands seem to render the indigestion of food not only difficult, but impossible. As regards the ability to take nutriment, the condition is analogous to that in which a temporary complete anorexia is caused by repletion of the stomach. It remains to be determined whether the inability to take certain kinds of food may not have a relation to the destruction of the gastric and the intestinal tubules separately. Considering the difference in function between these two sets of glands, it may be conjectured that, if the gastric tubules be alone affected, the anorexia will relate chiefly to the albuminoid or nitrogenous articles of diet; and the ability to ingest and digest fats, starch and sugar, remaining, emaciation or the loss of weight will not be marked—a fact observed in the case lately reported by Fenwick, and in the cases of idiopathic anæmia described by Addison. On the other hand, we may conjecture that, if the intestinal tubules be alone destroyed, the ability to injest and digest albuminoid substances may be retained, and the anorexia, together with the loss of digestive power, will relate to the non-nitrogenous principles of diet. In the latter case, it would be expected that emaciation should be more marked. But the changes occurring in the intestinal tubules, either with or without changes in the corresponding glands of the stomach, are yet to be studied.

